# Determinants of Attitude Toward End-of-Life Care Among Junior Physicians: Findings from a Nationwide Survey in Japan

**DOI:** 10.1089/pmr.2023.0004

**Published:** 2023-09-01

**Authors:** Yukiko Watanabe, Natsuki Kawashima, Yu Uneno, Soichiro Okamoto, Manabu Muto, Tatsuya Morita

**Affiliations:** ^1^ Faculty of Medicine, Okayama University Medical School, Okayama, Japan.; ^2^ Department of Palliative and Supportive Care, Tsukuba, Japan.; ^3^ Department of Therapeutic Oncology, Graduate School of Medicine, Kyoto University, Kyoto, Japan.; ^4^ Medical Corporation Teieikai Chiba Home Care Clinic, Chiba, Japan.; ^5^ Division of Supportive and Palliative Care, Seirei Mikatahara General Hospital, Hamamatsu, Japan.

**Keywords:** attitude toward care for the dying, end-of-life care, FATCOD score, palliative care

## Abstract

**Background:**

Physicians' attitudes can be critical in quality end-of-life care. However, the determinants of the attitudes and whether the attitudes can be modified remain unclear.

**Objectives:**

To investigate factors correlated with physicians' positive attitudes toward end-of-life care and whether these attitudes are modifiable through acquired factors (e.g., education or mentorship).

**Design:**

A nationwide survey was conducted in 300 institutions and selected randomly from 1037 clinical training hospitals in Japan.

**Participants:**

From each selected institution, two resident physicians of postgraduate year (PGY) 1 or 2 and two clinical fellows from PGY 3–5 were requested to answer the survey.

**Measurements:**

The primary outcome was the Frommelt Attitudes Toward the Care of the Dying (FATCOD) scale score. Factors (e.g., the respondents' age, sex, number of years of clinical experience, training environment, religion, and beliefs around death) were examined for correlation with FATCOD score.

**Results:**

In all, 198 physicians and 134 clinical fellows responded to the survey (response rate: 33.0% and 22.3%, respectively). Factors with the strongest correlation with FATCOD scores were mostly unmodifiable factors (e.g., being female and one's beliefs around death). Modifiable factors were also identified—number of patient deaths experienced, level of interest in palliative care, availability of support from senior mentors, and frequency of consultation with nonphysician medical staff.

**Conclusion:**

Physicians' attitudes toward end-of-life care correlate more strongly with nonmodifiable factors, but attitudes can be meaningfully improved via mentoring by senior physicians. Future studies are warranted to determine the effective interventions to foster positive attitudes among physicians involved in end-of-life care.

## Introduction

End-of-life care is defined as “whole person care to relieve various burdens, including physical and psychological distress and to improve quality of life for patients and their families whose prognosis is expected to be within six months.”^1^ A multidisciplinary approach is necessary to alleviate patients' physical, psychological, social, and spiritual distress.^2–4^ Furthermore, health care professionals (HCPs) are expected to meet the various needs of patients and their families regarding treatment plans and place of care and navigate complex discussions regarding life-prolonging treatments.^5–7^

Delivering quality end-of-life care depends on a combination of the skills, knowledge, and attitudes of HCPs.^5–7^ Of these, the attitudes of HCPs toward patients at end-of-life were revealed as especially important by previous studies. This is because a positive attitude is related to improvements in HCPs' confidence, behavior, and care quality, and increases HCPs' chances of remaining in the end-of-life care field.^8–11^ However, communication, end-of-life discussion with patients and family members, symptom alleviation, and death pronouncement are the most challenging components of end-of-life care among junior physicians.^12–15^ Moreover, a nationwide survey of bereaved families in Japan revealed that only 38% answered that the patient was experiencing little suffering and physical pain, and only 44% thought that the patient was able to receive satisfactory treatment.^16^ Regarding communication, only 30% indicated that there was sufficient communication between the patient and the physician on topics such as preferred place of death and life-prolonging treatments.^16^ Despite these difficulties and clinical challenges, physicians often do not receive sufficient education or training in providing end-of-life care and feel unprepared to care for terminally ill patients (e.g., only 20% of physicians responded that they “received sufficient education regarding palliative and end-of-life care,” and only 30% responded that they had “sufficient knowledge and skills regarding alleviation of symptoms of end-of-life patients” in Japan).^12–15,17,18^

These previous studies pose a question about whether attitudes toward end-of-life care can be modified through training and educational programs or whether they are unmodifiable in each person, influenced by their upbringing, religious beliefs, and beliefs about death. A mandatory five-week, 12-lecture educational program on palliative care in Sweden was effective in significantly improving nursing students' attitudes toward end-of-life care.^19^ Another study from Sweden revealed that nursing students' attitudes toward care of dying patients were positively correlated with earlier education.^20^ If physicians' attitudes toward end-of-life care can improve with training and support systems, these educational interventions are valuable, and more resources should be allocated to them. Although many studies have investigated this topic among nurses and other HCPs, studies targeting junior physicians to evaluate their attitudes toward end-of-life care are limited.^21–26^ Furthermore, whether these attitudes of junior physicians have modifiable properties remains unknown.

This study aimed to investigate factors that correlate with physicians' positive attitudes toward end-of-life care and to investigate whether attitudes are modifiable through acquired factors such as education or mentorship.

## Materials and Methods

This secondary analysis of a nationwide survey was administered to junior physicians in the departments of internal medicine and surgery at government-designated clinical training hospitals in Japan.^12^ The government-designated clinical training hospitals are required to satisfy the comprehensive criteria on size (300+beds, with 3000+ inpatient admissions per year), assignment of independent multiple clinical departments including emergency medicine and full-time employed mentor physicians, number of full-time employed physicians, and number of autopsies per year, among other conditions.^27^ Junior physician is defined as residents of postgraduate year (PGY) 1 or 2, and clinical fellows of PGY 3–5. This study was reviewed and approved by the Ethics Committee of Tsukuba Medical Center Hospital (Approval ID: 2022–014). The detailed aims and concept of the survey were explained in the documents given to the participants, and responding to the survey was deemed as consent to participate.

### Survey process

The survey process is described elsewhere.^12^ The participants were from 300 institutions selected randomly from 1037 government-designated clinical training hospitals in Japan. Two resident physicians and two clinical fellows at each institution were requested to respond to the survey (each include one internal and surgical junior physician, respectively). The paper-based survey was distributed in January 2020 using a self-administered anonymous questionnaire, and a reminder was sent in February.

### Content of the questionnaire

At the beginning of the survey, end-of-life care was defined in accordance with previous literature, as “whole person care to relieve various burdens including physical and psychological distress and to improve quality of life for patients and their families whose prognosis is expected to be within six months.”^1^ Furthermore, the participants were instructed to answer questions about terminally ill patients with cancer.

#### Frommelt Attitude Toward the Care of the Dying scale

Frommelt Attitude Toward the Care of the Dying (FATCOD) scale was developed to measure the care attitudes of medical practitioners toward dying patients.^28,29^ Originally developed for use by nurses, Form B was published for use by physicians and medical staff.^28,29^ The Japanese version has been shown to have reliability and validity based on this Form B.^30^ The FATCOD-B has two subscales (“Positive attitude toward caring for the dying patient” and “Perception of patient and family-centered care”); based on our research interest, the “Positive attitude toward caring for the dying patient” subscale was used for the survey.

#### Explanatory variable for the FATCOD

Based on a comprehensive literature search of previous studies and discussion among the researchers, variables that have already been shown to be associated with FATCOD were identified. These included the respondents' background (sex, marital status, religion, etc.), training environment, clinical experience (number of terminally ill patients they have been in charge of, number of sick people they have cared for in their personal lives, etc.).^10,21–26^ Furthermore, we used the 7-domain, 27-item Death Attitude Inventory developed by Hirai et al. to measure views of life and death that are unique to Japanese culture.^31^

### Statistical analysis

Descriptive statistics were performed for frequency, proportions, and confidence intervals (95% CIs). Cronbach's α was calculated for internal consistency. The normal distribution of continuous variables was confirmed visually and using the Shapiro–Wilk test.^32^ Univariate analyses using Pearson's correlation coefficient and independent *t* test were first performed to determine the association between each exposure factor and FATCOD score. Multiple linear regression based on statistical variable selection was performed to evaluate the most correlated factors. Subsequently, a sensitivity analysis was conducted. In the variable selection of the sensitivity analysis, we bundled some variables which may have similarity and interactions into one variable (e.g., the variable “years of clinical experience” was omitted due to possible interaction with “number of patient deaths experienced”) and excluded variables that may have a weak association (e.g., marital status). All analyses were conducted using the statistical package Stata (version 17.0).

## Results

### Participant characteristics

**FIG. 1.**
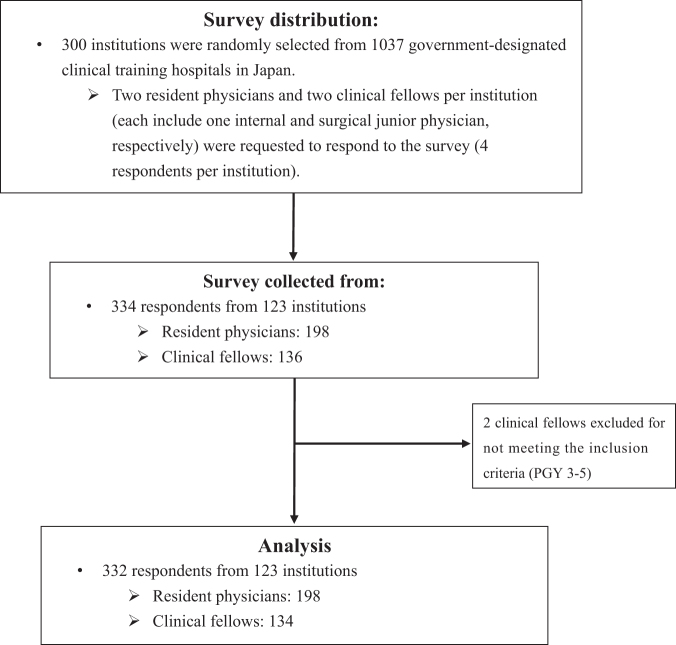
Diagram of the nationwide survey process.

The survey process is summarized in Figure 1. A total of 198 resident physicians (PGY 1 or 2) and 134 clinical fellows (PGY 3–5) were included in the study. The overall response rates were 33.0% and 22.3%, respectively. Details of the respondents' characteristics are described elsewhere.^12^

### FATCOD-B score

The overall mean FATCOD-B scores were 57.2 (95% CI: 56.2–58.2, *N* = 198) and 59.5 (95% CI: 58.1–60.8, *N* = 134) for resident physicians and clinical fellows, respectively. The corresponding Cronbach's α were 0.81, and 0.86, indicating score reliability.

### Univariate and multivariate analysis

Among junior physicians (resident physicians and clinical fellows), the univariate analysis revealed that FATCOD-B scores were positively associated with being female, number of years of clinical experience, number of patient deaths experienced (not restricted to cancer patients), higher levels of interest in palliative care, availability of support from senior mentors, and frequency of consultation with nonphysician medical staff (Table 1). Among the factors of the Death Attitudes Inventory, lower scores on “Death anxiety/fear,” “Death as release,” “Death avoidance,” and higher scores on “Sense of purpose in life” showed significant correlation with FATCOD-B scores. Similar trends were identified in the subgroup analysis of the univariate analysis, divided into resident physicians and clinical fellows groups (Supplementary Table S1). The multiple linear regression (adjusted *R*^2^ = 0.25) showed that being female, number of patients' deaths experienced, “Death avoidance,” and “Sense of purpose in life” have significant associations with FATCOD-B score (Table 2). Similar results were obtained in the subsequent sensitivity analysis (adjusted *R*^2^ = 0.24), where being female, number of patients' deaths experienced, “Death avoidance,” “Sense of purpose in life,” and “Death concern” were found to have significant associations with FATCOD-B score (Supplementary Table S2).

**Table 1.** Univariate Analysis of Correlation With FATCOD-B Score Using Pearson's Correlation Coefficient and an Independent *t* Test (*N* = 332)

		Pearson's r/mean FATCOD-B score ± SD	Coefficient	***p***
Age in years (mean ± SD)	28.2 ± 2.8	*r* = 0.06	0.2	0.249
Sex	Female [*N* = 95]	59.5 ± 6.6	2.0	0.028^*^
	Male [*N* = 236]	57.5 ± 7.6		
No. of years of clinical experience (mean ± SD)	2.5 ± 1.3	*r* = 0.16	0.9	0.004^*^
Marital status	Married [*N* = 82]	57.7 ± 8.9	0.5	0.592
	Not married [*N* = 250]	58.2 ± 6.8		
Has own family long-term care experience	Yes [*N* = 37]	57.9 ± 9.6	−0.2	0.880
	No [*N* = 295]	58.1 ± 7.0		
Has own family bereavement experience	Yes [*N* = 264]	58.2 ± 7.4	0.5	0.631
	No [*N* = 68]	57.7 ± 7.0		
Is religious	Yes [*N* = 76]	58.5 ± 8.0	0.5	0.618
	No [*N* = 256]	58.0 ± 7.2		
No. of patient deaths experienced	Less than 10 [*N* = 233]	57.2 ± 7.1	3.4	<0.001^*^
(Not restricted to cancer patients)	More than 11 [*N* = 94]	60.8 ± 7.4		
Interest in palliative care^**^	High [*N* = 299]	58.5 ± 7.3	4.2	0.002^*^
	Low [*N* = 32]	54.4 ± 7.2		
Has received education regarding palliative care	Yes [*N* = 297]	58.2 ± 7.6	0.4	0.773
	No [*N* = 34]	57.8 ± 5.3		
Has received clinical training in palliative care	Yes [*N* = 127]	57.8 ± 7.6	−0.5	0.584
	No [*N* = 204]	58.3 ± 7.2		
Has attended onsite palliative care seminar	Yes [*N* = 228]	58.4 ± 7.5	0.9	0.292
	No [*N* = 102]	57.5 ± 7.0		
Has support regarding end-of-life care from a mentor	Yes [*N* = 178]	59.0 ± 8.0	1.7	0.038^*^
	No [*N* = 147]	57.3 ± 6.5		
Frequency of palliative care consultation to expert teams^***^	High [*N* = 214]	58.3 ± 7.1	0.5	0.559
	Low [*N* = 118]	57.8 ± 7.8		
Frequency of consultation to nonphysician medical staff^***^	High [*N* = 268]	58.5 ± 7.3	2.0	0.048^*^
	Low [*N* = 63]	56.5 ± 7.2		
Involvement of other health care professionals at important	High [*N* = 294]	58.2 ± 7.4	0.5	0.702
meetings with patients and family^***^	Low [*N* = 37]	57.7 ± 7.0		
Attendance of death conference^***^	High [*N* = 146]	58.4 ± 7.6	0.4	0.603
	Low [*N* = 184]	58.0 ± 7.1		
Death Attitude Inventory				
Death anxiety (mean ± SD)	16.8 ± 6.5 (out of 20)	*r* = −0.21	−0.2	<0.001^*^
Death relief (mean ± SD)	11.1 ± 5.3 (out of 20)	*r* = −0.16	−0.2	0.004^*^
Death avoidance (mean ± SD)	10.8 ± 5.0 (out of 20)	*r* = −0.43	−0.6	<0.001^*^
Life purpose (mean ± SD)	16.2 ± 5.2 (out of 20)	*r* = 0.17	0.2	0.002^*^
Death concern (mean ± SD)	14.2 ± 4.8 (out of 20)	*r* = 0.06	0.1	0.291
Supernatural belief	9.3 ± 4.7 (out of 15)	*r* = −0.09	−0.1	0.096

^*^ Denotes statistical significance at *p* ≤ 0.05.

^**^ High: respondents who selected 1–3, Low: respondents who selected 4–5 on a scale of 1–5, 1 being most interested, 5 being not interested at all.

^***^ High: respondents who selected 1–3, Low: respondents who selected 4–5, on a scale of 1–5, 1 being Always, 5 being Never.

FATCOD, Frommelt Attitudes Toward the Care of the Dying; SD, standard deviation.

**Table 2.** Multiple Linear Regression Results Predicting FATCOD-B Score (*N* = 332, Adjusted *R*^2^ = 0.25)

	***β***	***p***
Sex	1.7	0.04^*^
Years of clinical experience	0.2	0.6
No. of patient deaths experienced (not restricted to cancer patients)	2.2	0.02^*^
Interest in palliative care	2.2	0.08
End-of-life care support by a mentor	−0.2	0.81
Frequency of consultation with nonphysician medical staff	0.9	0.37
Death Attitude Inventory		
Death anxiety/fear	−0.1	0.44
Death as release	−0.1	0.37
Death avoidance	−0.6	<0.001^*^
Sense of purpose in life	0.2	<0.001^*^

^*^ Denotes statistical significance at *p* ≤ 0.05.

## Discussion

Our survey attempted to identify factors that influence junior physicians' attitudes toward end-of-life care. While the multiple linear regression analysis revealed that nonmodifiable factors had a strong correlation with FATCOD scores, the univariate analysis showed that modifiable factors such as mentoring by senior physicians and discussion with nonphysician medical staff were significantly correlated with positive attitudes. This implies that positive attitudes toward caring for the dying can be achieved with the appropriate modes of intervention.

Multiple linear regression and the following sensitivity analysis revealed that the factors significantly associated with higher FATCOD-B scores were nonmodifiable. These factors include being female, number of years of clinical experience, and one's beliefs around death. Of the seven categories of the Death Attitudes Inventory, scores on “Death anxiety/fear,” “Death as release,” and “Death avoidance” were negatively associated with FATCOD-B scores, and scores on “Sense of purpose in life” were positively associated higher FATCOD-B scores. Years of clinical experience and number of patient deaths experienced are both significantly associated with higher FATCOD-B scores, which is in line with previous findings.^21–26^ The reason for years of clinical experience not being significant in the multivariate analysis may be that the association was weaker than those of other variables in the population of junior physicians. Knowing that these nonmodifiable factors strongly correlate with attitudes may help physicians become more self-aware of their predispositions and tendencies when caring for the dying. Furthermore, this knowledge may be considered when designing future educational or training programs by tailoring the content to meet the needs of the audience.

The modifiable factors that showed significant associations with higher FATCOD-B scores were number of patient deaths experienced, level of interest in palliative care, availability of support from senior mentors, and frequency of consultation with nonphysician medical staff regarding the care of end-of-life patients. As suggested by Laporte et al., more encounters with terminally ill patients, and deaths of patients may help ease physicians' anxiety and lack of confidence.^33^ Moreover, this is consistent with a previous report that the availability of attending physicians with whom junior physicians can easily consult was selected as the most favorable support measure to promote confidence about end-of-life care among junior physicians.^12^ This confirms the importance of having a close mentor–mentee relationship in an end-of-life care clinical setting for junior physicians. In the same study, the second most helpful support measure was the availability of staff and departments where junior physicians can discuss their mental burden and stress.^12^ These findings show the importance of providing a close support network to help ease junior physicians' emotional burden and stress during end-of-life care.

This study has some limitations. First, the response rate was low (22–33%), which may have resulted in a selection bias; in other words, physicians who are more interested in end-of-life care research may have been more willing to answer the survey. Further efforts are needed to improve the response rate, such as conducting electronic surveys and providing incentives. Nonetheless, this study elucidates the overall trends of the determinants of attitude toward end-of-life care among junior physicians, which can potentially help navigate future efforts to deliver effective support, including mentorship, while emphasizing clinical experiences. Second, while random sampling was performed to ensure representativeness of the facility, no concrete steps were taken to guarantee representativeness of respondents within each facility. Clerical staff in each facility were responsible for distributing the survey to the physicians, but no action was requested to ensure the representativeness of the background characteristics of the physicians, such as their specialty. Third, despite the statistically significant difference, Pearson's *r*-values were very small (Table 1). This is consistent with previous studies investigating the association with FATCOD-B, suggesting there are limited factors that are strongly correlated with end-of-life care attitudes.^34^ Finally, some relevant factors were not included in the questionnaire, such as whether being an internal or junior surgical physician is a relevant factor for attitudes toward end-of-life care; however, our main interest was factors related to all attitudes of junior physicians. For further research, it is important to include comprehensive relevant factors in the questionnaire.

## Conclusions

This study revealed that attitudes toward caring for the dying are largely determined by nonmodifiable factors such as gender and one's beliefs around death. Furthermore, we identified potential intervention methods (e.g., mentoring by senior physicians and consultation with nonphysician medical staff). Future studies should be conducted to determine other effective interventions to foster positive attitudes among physicians involved in end-of-life care.

## Supplementary Material

Supplemental data

Supplemental data

## Data Availability

The datasets analyzed during the current study are available from the corresponding author on reasonable request.

## Abbreviations Used

CI: confidence interval
FATCOD: Frommelt Attitudes Toward the Care of the Dying
HCPs: health care professionals
PGY: postgraduate year
