# Supplementary material for: Determinants of Attitude Toward End-of-Life Care Among Junior Physicians: Findings from a Nationwide Survey in Japan
Source: Palliat Med Rep. 2023 Sep 1;4(1):257–63. doi: 10.1089/pmr.2023.0004 (PMC10507924; doi:10.1089/pmr.2023.0004)
Supplement: Supplemental data [file Suppl_TableS1.docx]

| Additional Table 1. Univariate analysis of correlation with FATCOD-B score  using Pearson’s correlation coefficient and an independent *t* test (by PGY) | | | | | | | | | | |
| --- | --- | --- | --- | --- | --- | --- | --- | --- | --- | --- |
|  | Resident physicians (PGY1-2) |  |  |  |  | Clinical fellows (PGY3-5) |  |  |  | |
| Characteristics | | Pearson's r /  Mean FATCOD-B score ± SD | Coef. | P-value |  |  | Pearson's r /  Mean FATCOD-B score ± SD | Coef. | P-value | |
| Total | [N=198] | 57.2±6.9 |  |  |  | [N=134] | 59.5±7.8 |  |  | |
| Age in years (mean ± SD) | 27.5±2.9 | 0.006 | 0.0 | 0.932 |  | 29.2±2.4 | 0.04 | 0.1 | 0.617 | |
| Sex | Female [N=48] | 58.0±6.3 | 1.1 | 0.337 |  | Female [N=47] | 61.0±6.6 | 2.4 | 0.089 | |
|  | Male [N=149] | 56.9±7.1 |  |  |  | Male [N=87] | 58.6±8.2 |  |  | |
| Number of years of clinical experience (mean±SD) | 1.6±0.5 | -0.04 | -0.6 | 0.533 |  | 3.9±0.8 | 0.13 | 1.3 | 0.126 | |
| Marital status | Married [N=37] | 56.4±8.3 | 1.0 | 0.451 |  | Married [N=45] | 58.8±9.3 | 1.0 | 0.496 | |
|  | Not married [N=161] | 57.4±6.6 |  |  |  | Not married [N=89] | 59.8±6.9 |  |  | |
| Has own family long-term care experience | Yes [N=24] | 57.5±7.1 | 0.4 | 0.802 |  | Yes [N=13] | 58.7±13.5 | -0.9 | 0.709 | |
|  | No [N=174] | 57.2±6.9 |  |  |  | No [N=121] | 59.5±7.0 |  |  | |
| Has own family bereavement experience | Yes [N=164] | 57.3±6.7 | 0.7 | 0.567 |  | Yes [N=100] | 59.7±8.4 | 0.8 | 0.617 | |
|  | No [N=34] | 56.6±8.1 |  |  |  | No [N=34] | 58.9±5.7 |  |  | |
| Is religious | Yes [N=46] | 57.5±7.0 | 0.4 | 0.746 |  | Yes [N=30] | 60.0±9.3 | 0.7 | 0.667 | |
|  | No [N=152] | 57.1±6.9 |  |  |  | No [N=104] | 59.3±7.3 |  |  | |
| Number of patients' deaths experienced | Less than 10 [N=174] | 57.1±6.8 | 2.0 | 0.229 |  | Less than 10 [N=59] | 57.4±8.0 | 3.6 | 0.007* | |
| (not restricted to cancer patients) | More than 11 [N=20] | 59.1±7.7 |  |  |  | More than 11 [N=74] | 61.0±7.3 |  |  | |
| Interest in palliative care** | High [N=174] | 57.6±6.7 | 3.4 | 0.026* |  | High [N=125] | 59.8±7.8 | 5.0 | 0.061 | |
|  | Low [N=23] | 54.2±7.8 |  |  |  | Low [N=9] | 54.8±5.9 |  |  | |
| Has received education regarding palliative care | Yes [N=175] | 57.2±7.1 | -0.7 | 0.655 |  | Yes [N=122] | 59.6±8.0 | 2.0 | 0.404 | |
|  | No [N=22] | 57.9±5.9 |  |  |  | No [N=12] | 57.7±4.0 |  |  | |
| Has received clinical training in palliative care | Yes [N=67] | 56.3±6.3 | -1.3 | 0.196 |  | Yes [N=60] | 59.5±8.6 | 0.1 | 0.922 | |
|  | No [N=130] | 57.6±7.2 |  |  |  | No [N=74] | 59.4±7.1 |  |  | |
| Has attended onsite palliative care seminar | Yes [N=124] | 57.4±7.0 | 0.5 | 0.624 |  | Yes [N=104] | 59.6±7.9 | 0.6 | 0.699 | |
|  | No [N=73] | 56.9±6.9 |  |  |  | No [N=29] | 59.0±7.4 |  |  | |
| Has support regarding end-of-life care from a mentor | Yes [N=98] | 57.9±7.1 | 1.3 | 0.192 |  | Yes [N=80] | 60.2±8.8 | 1.8 | 0.195 | |
|  | No [N=95] | 56.6±6.9 |  |  |  | No [N=52] | 58.4±5.7 |  |  | |
| Frequency of palliative care consultation to expert | High [N=116] | 57.1±6.1 | -0.3 | 0.768 |  | High [N=98] | 59.7±7.9 | 1.0 | 0.523 | |
| teams*** | Low [N=82] | 57.4±8.0 |  |  |  | Low [N=36] | 58.8±7.4 |  |  | |
| Frequency of consultation to non-physician | High [N=147] | 57.5±6.6 | 0.9 | 0.451 |  | High [N=121] | 59.8±8.0 | 3.7 | 0.098 | |
| medical staff*** | Low [N=50] | 56.6±7.8 |  |  |  | Low [N=13] | 56.1±4.6 |  |  | |
| Involvement of other health care professionals at | High [N=173] | 57.2±6.9 | -0.5 | 0.725 |  | High [N=121] | 59.7±7.9 | 2.0 | 0.390 | |
| important meetings with patients and family*** | Low [N=24] | 57.7±7.3 |  |  |  | Low [N=13] | 57.7±6.7 |  |  | |
| Attendance of death conference*** | High [N=86] | 58.0±6.9 | 1.4 | 0.159 |  | High [N=60] | 58.9±8.6 | -1.1 | 0.436 | |
|  | Low [N=110] | 56.6±7.0 |  |  |  | Low [N=74] | 59.9±7.0 |  |  | |
| **Death Attitude Inventory** |  |  |  |  |  |  |  |  |  | |
| Death anxiety (mean±SD) | 16.9±6.6 (out of 20) | -0.16 | -0.2 | 0.022* |  | 16.5±6.3 | -0.27 | -0.3 | 0.002* | |
| Death relief (mean±SD) | 11.3±5.4 (out of 20) | -0.11 | -0.1 | 0.115 |  | 10.9±5.2 | -0.21 | -0.3 | 0.015* | |
| Death avoidance (mean±SD) | 10.8±5.1 (out of 20) | -0.36 | -0.5 | <0.001* |  | 10.8±4.9 | -0.53 | -0.8 | <0.001* | |
| Life purpose (mean±SD) | 16.2±5.2 (out of 20) | 0.27 | 0.4 | <0.001* |  | 16.2±5.2 | 0.04 | 0.1 | 0.680 | |
| Death concern (mean±SD) | 14.3±4.8 (out of 20) | 0.07 | 0.1 | 0.300 |  | 14.1±4.8 | 0.04 | 0.1 | 0.617 | |
| Supernatural belief (mean±SD) | 9.1±4.6 (out of 15) | -0.11 | -0.2 | 0.134 |  | 9.8±4.8 | -0.1 | -0.2 | 0.248 | |
| SD: standard deviation; | | | | | | | | | |  |
| *Denotes statistical significance at P ≤ 0.05 | | | | | | | | | |  |
| **High: respondents who selected 1~3, Low: respondents who selected 4~5 on a scale of 1~5, 1 being most interested, 5 being not interested at all | | | | | | | | | |  |
| ***High: respondents who selected 1~3, Low: respondents who selected 4~5, on a scale of 1-5, 1 being Always, 5 being Never | | | | | | | | | |  |
