# Supplementary material for: Determinants of Attitude Toward End-of-Life Care Among Junior Physicians: Findings from a Nationwide Survey in Japan
Source: Palliat Med Rep. 2023 Sep 1;4(1):257–63. doi: 10.1089/pmr.2023.0004 (PMC10507924; doi:10.1089/pmr.2023.0004)
Supplement: Supplemental data [file Suppl_TableS2.docx]

| Additional Table 2. Multiple linear regression results predicting FATCOD-B score (*N*=332, Adjusted R-squared = 0.24) | |
| --- | --- |
| \|  \|  \| Coef. \| P-value \| \| --- \| --- \| --- \| --- \| \| Age in years (mean ± SD) \| 28.2 ± 2.8 \| 0.0 \| 0.850 \| \| Sex \| Female [N=95] \| 1.7 \| 0.039* \| \|  \| Male [N=236] \|  \|  \| \| Is religious \| Yes [N=76] \| 0.2 \| 0.834 \| \|  \| No [N=256] \|  \|  \| \| Number of patients' deaths experienced \| Less than 10 [N=233] \| 2.7 \| 0.002* \| \| (not restricted to cancer patients) \| More than 11 [N=94] \|  \|  \| \| Interest in palliative care** \| High [N=299] \| 2.1 \| 0.099 \| \|  \| Low [N=32] \|  \|  \| \| Has received education regarding palliative care \| Yes [N=297] \| 0.1 \| 0.953 \| \|  \| No [N=34] \|  \|  \| \| Has support regarding end-of-life care by mentor \| Yes [N=178] \| -0.2 \| 0.754 \| \|  \| No [N=147] \|  \|  \| \| Frequency of palliative care consultation to expert teams*** \| High [N=214] \| 0.3 \| 0.680 \| \|  \| Low [N=118] \|  \|  \| \| **Death Attitude Inventory** \|  \|  \|  \| \| Death anxiety \| Mean 16.8 (out of 20) \| -0.1 \| 0.307 \| \| Death relief \| Mean 11.1 (out of 20) \| -0.1 \| 0.221 \| \| Death avoidance \| Mean 10.8 (out of 20) \| -0.6 \| <0.001* \| \| Life purpose \| Mean 16.2 (out of 20) \| 0.2 \| 0.018* \| \| Death concern \| Mean 14.2 (out of 20) \| 0.2 \| 0.020* \| \| Supernatural belief \| Mean 9.3 (out of 15) \| -0.1 \| 0.311 \| | |
| SD: standard deviation; |  |
| *Denotes statistical significance at P ≤ 0.05 |  |
| **High: respondents who selected 1~3, Low: respondents who selected 4~5 on a scale of 1~5, 　　1 being most interested, 5 being not interested at all |  |
| ***High: respondents who selected 1~3, Low: respondents who selected 4~5, on a scale of 1-5, 　1 being Always, 5 being Never |  |
